# Supplementary material for: Conditional chemoconnectomics (cCCTomics) as a strategy for efficient and conditional targeting of chemical transmission
Source: eLife. 2024 Apr 30;12:RP91927. doi: 10.7554/eLife.91927 (PMC11060718; doi:10.7554/eLife.91927)
Supplement: Figure 3—source data 3. [file elife-91927-fig3-data3.zip › Figure 3 source data3 part 1.zip!/Figure 3-Source data 3-part1/1081-1-CRISPResso_Report_8zosk6yj.zip!/CRISPResso_on_1081_2/2b.Nucleotide_percentage_quilt_around_sgRNA_tttctggactacagtcccca.pdf]

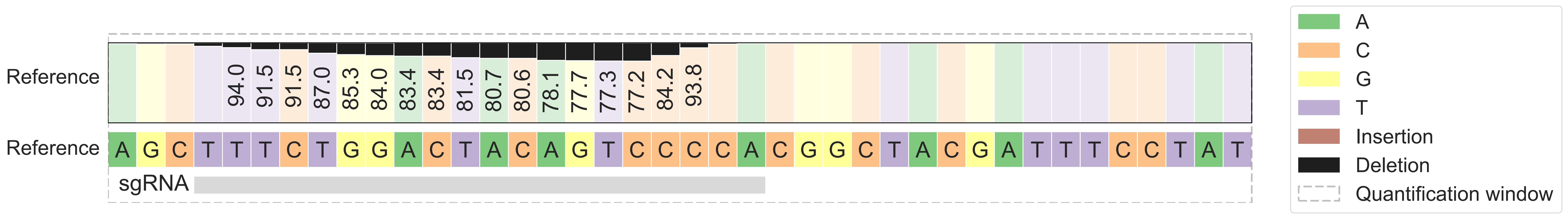
Reference
94.0
91.5
91.5
87.0
85.3
84.0
83.4
83.4
81.5
80.7
80.6
78.1
77.7
77.3
77.2
84.2
93.8
Reference
A G C T T T C T G G A C T A C A G T C C C C A C G G C T A C G A T T T C C T A T
sgRNA
A
C
G
T
Insertion
Deletion
Quantification window
